# PUBLIC POLICY AND AGING

**DOI:** 10.1093/geroni/igae098.0909

**Published:** 2024-12-31

**Authors:** Michael Lepore

**Affiliations:** University of Massachusetts Amherst, Amherst, Massachusetts, United States

## Abstract

N/A

